# Current Understanding of Hydrogel for Drug Release and Tissue Engineering

**DOI:** 10.3390/gels8050301

**Published:** 2022-05-15

**Authors:** Lanjie Lei, Yujing Bai, Xinyun Qin, Juan Liu, Wei Huang, Qizhuang Lv

**Affiliations:** 1 College of Biology & Pharmacy, Yulin Normal University, Yulin 537000, China; leilanjie1988@163.com (L.L.); yujingbai2022@163.com (Y.B.); 18867024673@163.com (X.Q.); liujuan5269@163.com (J.L.); huangwei@ylu.edu.cn (W.H.); 2 Key Laboratory of System Bio-Medicine of Jiangxi Province, Jiujiang University, Jiujiang 332000, China; 3 Guangxi Key Laboratory of Agricultural Resources Chemistry and Biotechnology, Yulin 537000, China

**Keywords:** responsive hydrogels, drug release, tissue engineering, synthetic polymer, natural polymer

## Abstract

Due to their good absorption, satisfactory biocompatibility, and high safety, hydrogels have been widely used in the field of biomedicine, including for drug delivery and tissue regeneration. In this review, we introduce the characteristics and crosslinking methods of natural and synthetic hydrogels. Then, we highlight the design and principle of intelligent hydrogels (i.e., responsive hydrogels) used for drug release. Moreover, we introduce the application of the application of hydrogels in drug release and tissue engineering, and the limitations and research directions of hydrogel in drug release and tissue engineering are also considered. We hope that this review can provide a reference for follow-up studies in related fields.

## 1. Introduction

Hydrogel-based, sustained-release drug carriers are an emerging drug delivery system (DDS). By using materials that are nontoxic, have a good biocompatibility, and are biodegradable as carriers or media, the DDS can be chemically or physically bound to the drug to create the corresponding drug dosage form. A DDS enters the body via chemical diffusion or penetration; thus, drugs can be released slowly and continuously into the human body at a stable rate and an appropriate concentration to improve their efficacy, reduce the drug dosage, and provide an optimal therapeutic effect [1]. As one of the important components of a sustained-release system, the controlled-release drug carrier plays a very important role in the curative effect of the drug. Different kinds of controlled-release drug carriers or the same carrier under different conditions have different delivery characteristics and slow-release performance; thus, the development of targeted slow-release drug carriers is very important [2]. In recent years, a wide variety of hydrogels have been used in biomedical applications, including drug delivery and tissue regeneration. The hydrogel materials used for drug release can be divided into natural polymer materials (such as chitosan, alginate, cyclodextrin, and collagen) and synthetic polymer materials (such as *N*-isopropylacrylamide (NIPAM), acrylamide, polyvinyl alcohol (PVA), and polyethylene glycol (PEG)) [3]. Hydrogels with various properties, such as water absorption, swelling, and degradation effects, can greatly improve the utilization rate of drugs and help to control their release [4]. Because of their advantageous properties, hydrogels based on drug controlled-release systems in tissue engineering are the most notable. In this review, we introduce the research progress made on the application of hydrogels as sustained-release drug carriers in tissue engineering. Firstly, we introduce some raw materials that are commonly used for preparing hydrogels, including natural materials and synthetic materials, and also briefly introduce crosslinking methods that are commonly used for preparing hydrogels from various materials. Secondly, we summarize the common characterization methods of hydrogel materials, as it is often necessary to conduct characterization research before the application of hydrogel materials, and the mechanisms of hydrogel materials and their applications are discussed. Lastly, we summarize the current understanding of intelligent (responsive) hydrogels and their applications in tissue engineering. In particular, the limitations of hydrogels in sustained-release drug carriers and tissue engineering are reviewed and discussed, which may be helpful for subsequent research using hydrogels as sustained-release carriers for tissue repair.

## 2. Classification of Hydrogels

**Figure 1:**
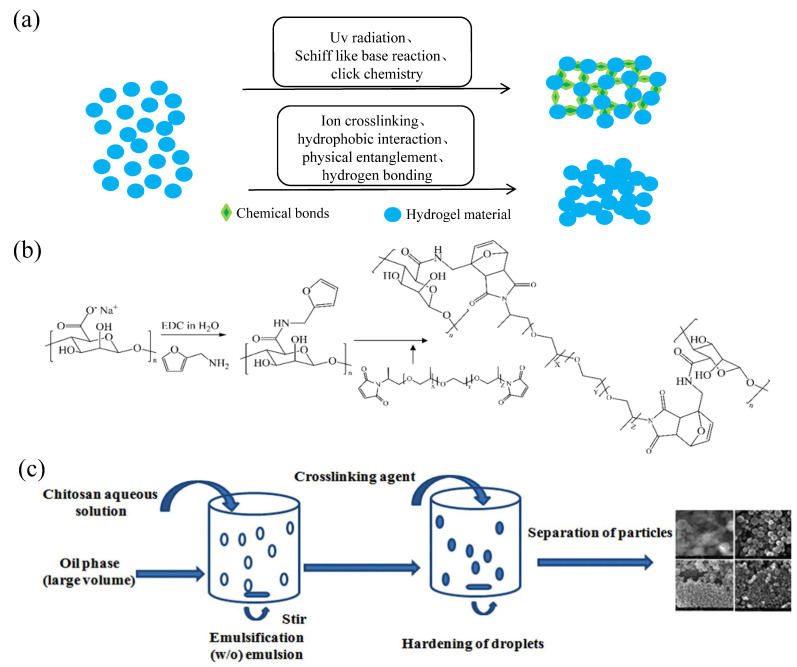
Crosslinking methods of hydrogels and preparation technologies of partial hydrogels. (**a**) Chemical and physical crosslinking of hydrogels. (**b**) Modification of alginate by furfurylamine and hydrogel formation by crosslinking with bismaleimide. Reprinted with permission from Ref. [10]. Copyright 2019, Fernando, I. P. S. (**c**) Schematic diagram of chitosan particle system prepared by emulsification crosslinking method. Reprinted with permission from Ref. [12]. Copyright 2011, Dash, M.

According to their source, the materials used to prepare hydrogels can be divided into natural materials and synthetic materials. Natural hydrogel materials are from the extracts of animals and plants, including collagen, chitosan, hyaluronic acid, alginate, chitin, gelatin, and cellulose, and have good biocompatibility and biodegradable properties; thus, they are widely used in the field of biomedical applications. Synthetic hydrogel materials, such as polyacrylic acid, polyacrylic acid salt (ammonium), polyacrylamide and its derivatives, PVA, polypeptide, and polyethylene oxide (PEO), can be industrially produced and chemically modified, and they have the advantages of precision and control [5]. However, compared with natural hydrogels, their biosafety and biodegradability are poorer due to the addition of initiators and crosslinking agents during the preparation process [6]. In addition, hydrogels can be divided into chemically crosslinked hydrogels and physically crosslinked hydrogels according to their crosslinking methods. Chemically crosslinked hydrogels are made by dissolving substances containing reactive groups with crosslinking agents in water under certain reaction conditions. Their structure is relatively stable, and they are often referred to as “permanent” gels. Common chemical crosslinking methods include ultraviolet radiation crosslinking, click chemical crosslinking, and Schiff base crosslinking. Physical crosslinking refers to hydrogels produced by crosslinking on the basis of noncovalent bonds, such as hydrogen bonds, ionic bonds, and van der Waals forces, whose state may alter with changes in the external conditions. Therefore, physically crosslinked hydrogels are often referred to as “reversible” hydrogels. Common physical crosslinking modes include ion crosslinking, hydrophobic interaction, physical entanglement, and hydrogen bonding [7] (Figure 1a).

### 2.1. Natural Hydrogels

Sodium alginate (SA) is a natural polysaccharide derived from brown seaweed that is hygroscopic, soluble in water, and can produce sticky latex. Because SA has good biocompatibility with organisms and does not cause allergy or inflammation, high-purity sodium alginate can exist in organisms; therefore, SA is often used to make sustainable-release carriers for drugs. In terms of material degradation, SA can be degraded by acid hydrolysis, enzymolysis, radiation degradation, and the addition of reducing substances [8,9]. The commonly used crosslinking methods of SA hydrogel preparations include enzyme crosslinking, covalent crosslinking, and ion crosslinking. Ion crosslinking is physical crosslinking. For example, a mixed SA–calcium salt gel can be formed after the SA hydrogel is combined with divalent cations such as calcium. However, the crosslinking process is rapid and difficult to control; hence, the hydrogel prepared is uneven and has poor mechanical properties [9]. Covalent crosslinking refers to chemical crosslinking and includes hydroxyl, carboxyl, Schiff base, and double-bond crosslinking. Although SA hydrogels with better performance can be prepared via chemical crosslinking, chemical crosslinking needs to be performed under harsh conditions, where toxic substances may be introduced, reducing their biocompatibility [10,11] (Figure 1b).

Chitosan is a kind of natural amino polysaccharide isolated from the shells of arthropods. Under weakly acidic conditions, some of the amino groups can be protonated and converted into quaternary ammonium salt, thus showing antibacterial activity. At the same time, due to its good biocompatibility, low toxicity, and hydrophilicity, it is also an ideal material for preparing hydrogels [11,12] (Figure 1c). In terms of biocompatibility, studies have shown that chitosan materials can cause nonspecific inflammatory reactions in the early stage of implantation, but as time goes by, the inflammatory reactions caused by chitosan materials gradually disappear. In terms of degradation, chitosan materials can be degraded by enzymatic hydrolysis, oxidation, acid hydrolysis, and other methods. The preparation methods of chitosan hydrogels include physical, chemical, and enzyme crosslinking methods, among which physical and chemical crosslinking methods are the most widely used. The physical crosslinking methods can be subdivided into ion crosslinking, hydrogen bond crosslinking, and polyelectrolyte complex crosslinking. The characteristics of the physical crosslinking preparation of chitosan hydrogels are that there is no need to add a chemical crosslinking agent; thus, there are few impurities that can be reused. Commonly used chemical crosslinking methods for the preparation of chitosan hydrogels include amidation reactions, purple diplomatic linking reactions, Schiff base reactions, and enzymatic crosslinking. Compared with hydrogels prepared by physical crosslinking, chemically crosslinked chitosan hydrogels have stronger mechanical strength and stability, as well as better performance in drug release and tissue engineering [13,14].

Hyaluronic acid (HA), a natural linear macromolecular polysaccharide, widely exists in organisms [15]. Due to its unique properties, including good biocompatibility (such as low cytotoxicity and weak immune response) and biodegradability (such as physical, chemical, and enzyme degradation methods), HA has also been widely used in biomedical fields in applications such as drug carriers and tissue engineering [16]. The preparation methods of HA hydrogels include physical and chemical crosslinking methods. HA can form hydrogels by temporary physical crosslinking through intermolecular physical forces, such as hydrophobicity, hydrogen bonding, and electrostatic interactions, but hydrogels prepared by intermolecular forces are very unstable. However, chemically crosslinked hydrogels made using the hydroxyl and carboxyl groups of HA and bifunctional small-molecule crosslinkers have better mechanical properties and quality. Commonly used crosslinking agents include glutaraldehyde, epoxy compounds, multifunctional hydrazides, and 1-ethyl-3-(3-dimethylaminopropyl) carbodiimide hydrochloride (EDC) [17].

**Figure 2:**
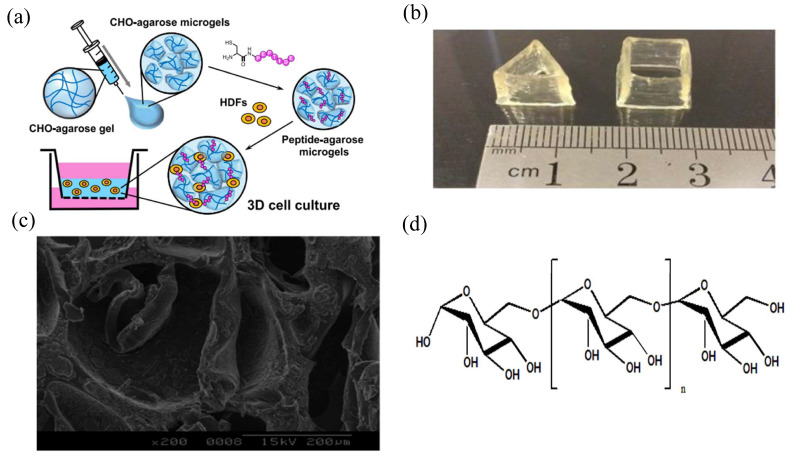
Shape and internal structure of parts of hydrogels. (**a**) Schematic illustration of the preparation of the peptide-agarose microgel scaffold for three-dimensional (3D) cell culture. Reprinted with permission from Ref. [18]. Copyright 2020, Yamada, Y. (**b**) Different forms of carrageenan composite hydrogel. Reprinted with permission from Ref. [23]. Copyright 2017, Liu, S. (**c**) Internal structure diagram of chondroitin sulfate composite hydrogel. Reprinted with permission from Ref. [27]. Copyright 2005, Dawlee, S. (**d**) Structure of dextran. Reprinted with permission from Ref. [28]. Copyright 2020, Chen, F.

Agarose, the main component of galactan, is a natural plant polysaccharide with the advantages of nontoxicity, low material cost, and a simple gel-forming method. It also has good temperature sensitivity, is generally soluble in water at 90 °C, and can form a stable hydrogel structure when the temperature drops to 40 °C. In addition, the hydrogel prepared with agarose as raw material can be degraded by enzyme degradation and chemical degradation, and it not only does not cause an immune response, but also promotes the growth of tissue cells in the body. Therefore, it is widely used in biomedical fields such as cell therapy, sustained drug release, and tissue engineering [18] (Figure 2a). The crosslinking method of agarose is similar to that of other natural hydrogels. Agarose hydrogels can also be formed through physical crosslinking and chemical crosslinking methods, such as van der Waals forces, hydrophobic forces, and hydrogen bond forces. Chemical crosslinking methods, such as click chemical reactions, Schiff base reactions, and Michael addition reactions, show better performance than physical crosslinking [19].

Carrageenan, also known as carrageenan glue, is a hydrophilic polysaccharide extracted from seaweed horseradish. It generally exists as white or light-brown granules or powder, and it is odorless and tasteless [20]. In addition to having rich sources, carrageenan is also safe, bioinert, environmentally friendly, nontoxic, and biodegradable, and it has been widely used in the food and medical fields [21]. In the field of controlled-release drug carriers, hydrogels can be prepared from carrageenan through physical, chemical, and radiation crosslinking. Pourjavdi et al. [22] introduced gold nanoparticles into a mixture containing carrageenan, chitosan, and polyisopropylacrylamide to form physically crosslinked hydrogels. Hydrogels prepared in this way have temperature sensitivity and good biocompatibility. Liu et al. [23] used KCl as an ionic crosslinking agent of carrageenan to prepare hydrogels with high toughness, recovery, and self-healing ability (Figure 2b). Kamalesh Prasad et al. [24] obtained a blended hydrogel of carrageenan and polyvinylpyrrolidone and found that its strength and swelling performance were significantly improved.

Chondroitin sulfate (CS), a natural acidic mucopolysaccharide, exists in the cartilage tissues of mammals and has anti-arthritis properties, the ability to regulate body immunity (including an increase in the weight of mouse immune organs, the function of mouse immune cells, and the secretion capacity of IFN-γ of the body), anti-coagulation properties, etc. [25]. In addition, CS can be degraded by enzymatic and chemical methods. In the field of tissue engineering, CS hydrogels can promote the formation and differentiation of cartilage and provide a biomimetic microenvironment without growth factors for the growth of chondrocytes and the regeneration of cartilage tissues. In addition, CS hydrogels can also meet the mechanical requirements for cartilage tissue repair in the body [26]. CS hydrogels can be prepared by physical and chemical crosslinking methods, such as hydrogen bond forces, hydrophobic forces, and van der Waals forces. Chemical crosslinking methods involve the use of a Schiff base reaction or glutaraldehyde as the crosslinking agent [19,27] (Figure 2c).

Glucan, also known as dextran, is a polysaccharide produced by bacteria such as *Candida intestinalis* through sucrose fermentation. Due to different fermentation strains and fermentation conditions, different glucans have certain differences in structure and molecular weight, but generally have water solubility [28] (Figure 2d). In the field of tissue engineering, glucan has the advantages of good histocompatibility, low inflammatory response, and no obvious tissue lesions; thereby, glucan hydrogels can be used as sustained-release carriers of drugs, which can realize the controlled release of drugs and can also destroy the spatial structure of hydrogels through the degradation of glucanase in the body to accelerate the release of drugs [29]. Glucan hydrogels can be prepared by physical and chemical crosslinking. Although physical crosslinking can avoid the toxicity of the crosslinking agent and the possible interaction between the crosslinking agent and the drug such that the activity of the drug can be preserved to the maximum extent, it also has the disadvantage of having the low strength of the hydrogels prepared. Although glucan hydrogels prepared by chemical crosslinking involve toxic crosslinking agents, the hydrogels produced by the chemical crosslinking method have better mechanical strength; hence, they are widely used. The crosslinking agents commonly used in the chemical crosslinking of glucan hydrogels include epichlorohydrin, metaphosphate, and borax [30].

**Figure 3:**
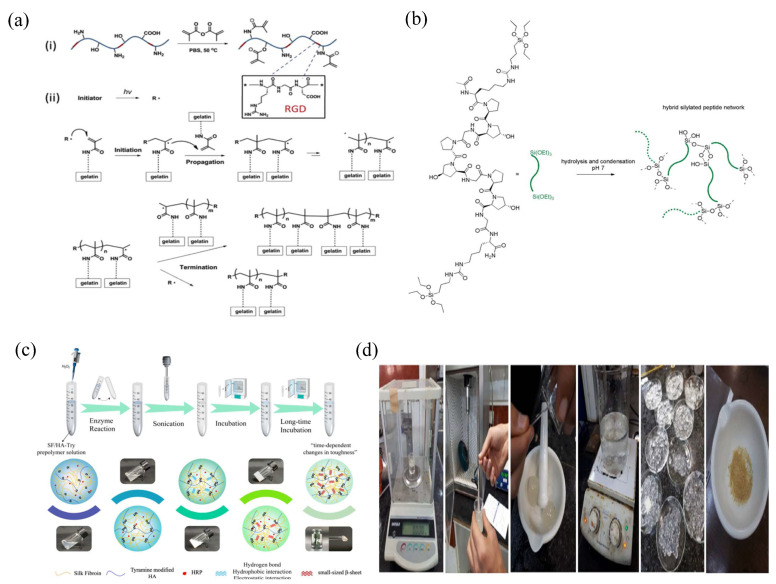
Preparation flow chart diagram of partial hydrogels. (**a**) Scheme for preparation of photo-crosslinked GelMA hydrogel ("*" in (i) denotes the left and right substituents of RGD; The "*" in (ii) stands for free radicals initiated by photocrosslinking agents). Reprinted with permission from Ref. [33]. Copyright 2022, Yue, K. (**b**) Bisilylated hybrid peptide as precursor for a cell-containing, collagen-inspired chemical hydrogel. Reprinted with permission from Ref. [36]. Copyright 2019, Echalier, C. (**c**) Schematic illustration of the preparation of the SF–HA composite hydrogels. Reprinted with permission from Ref. [39]. Copyright 2021, Qu, X.H. (**d**) Steps of synthesis of hydrogels containing starch by ultrasound (left to right). Reprinted with permission from Ref. [50]. Copyright 2021, Ebrahimi, R.

Gelatin, a natural biopolymer material derived from collagen, has the advantages of biodegradability (such as chemical or enzymatic degradation methods), good biocompatibility (for example, there was no redness, swelling, and exudation at the implanted site and no obvious inflammatory reaction was found at the site one week after surgery when gelatin microspheres were implanted subcutaneously in mice), and low cost, with a wide range of applications in the field of biomedicine, such as being used as a capsule material, matrix, and sustained-release carrier of drugs [31]. Among them, gelatin hydrogel, which can be used as a sustained-release carrier of drugs, has good water retention and viscoelasticity. After being dehydrated by applying pressure within a certain range, it can absorb water and swell again [32]. Current studies have shown that gelatin hydrogels can be prepared by chemical crosslinking, temperature crosslinking, photo-crosslinking, and enzyme crosslinking. However, due to the influence of various conditions, such as ambient temperature, the properties of hydrogels prepared by temperature crosslinking and photo-crosslinking are not ideal. Although gelatin hydrogels prepared by the chemical crosslinking agent glutaraldehyde have good effects, there are some disadvantages, such as the potential toxicity and biocompatibility of glutaraldehyde. However, hydrogels prepared using gelatin and methacrylic anhydride under certain conditions have a more stable performance [33] (Figure 3a).

Collagen, a structural protein of the extracellular matrix (ECM), is widely found in the skin, connective tissue, bone, and cartilage of vertebrates. Because collagen is derived from organisms, its immunogenicity is low, and it will not cause strong inflammation and immune response when applied to body tissues [34]. Collagen hydrogel has a structure similar to the ECM that is closer to a natural biological tissue structure and can be degraded by collagenase in the body; thus, it can give full play to its biological function. Currently, the methods available for gelatin hydrogel preparation include collagen self-assembly and chemical crosslinking [35]. Collagen self-assembly is a hydrogel formed by the self-assembly of collagen fibers, which are connected end-to-end in a quarter dislocation of collagen molecules, whereas the solvent in the solution is bound to its interior so that it cannot flow freely. This method mainly involves the preparation of hydrogels by noncovalent action; thus, its performance is poor [36]. Chemically crosslinked collagen hydrogels are formed through covalency. For example, small-molecule aldehydes and epoxides are commonly used as crosslinking agents, and the collagen hydrogels prepared have good elasticity but poor softness [36] (Figure 3b).

Silk fibroin (SF) is a protein existing in silk that is very similar to collagen in the body, therefore SF has good histocompatibility and low immunogenicity, is not able to easily cause an immune response of the body, and has certain biodegradability (it is usually degraded to amino acids or oligopeptides). In addition, its degradation products have no side-effects on the body and have nutrition and repair functions for surrounding tissues; thus, they are widely used in the field of tissue engineering [37]. SF hydrogels can be made via physical and chemical crosslinking. The physical crosslinking mechanism uses the sensitivity of the silk protein to molecular conditions such as pH value, shear, and vibration environment, inducing the formation of the beta folding structure to form a hydrogel. The commonly used physical crosslinking methods have a higher temperature and an adjustable pH value involving cyclone processing or ultrasonic processing. Although the strength of SF hydrogels achieved by physical crosslinking is high, they have some disadvantages, such as a long crosslinking time and a brittle texture. The mechanism of chemical crosslinking to prepare SF hydrogels involves the polymer chains in the formation of hydrogels being crosslinked through covalent bonds. Commonly used chemical crosslinking methods include crosslinking agents, light, and enzymes [38,39] (Figure 3c).

Sericin, like SF, is a protein existing in silk that is wrapped in the outer layer of SF and has a protective effect on SF. The obvious difference between SF and sericin is that SF can only swell in water but not dissolve, whereas sericin can dissolve in hot water [40]. As research on sericin has deepened, it has shown great advantages as a biomaterial due to its advantages of a stable source, strong processability, hydrophilicity, low immunogenicity, promotion of cell proliferation, inhibition of tyrosinase activity, and controllable degradation [41]. In the field of sustained-release drug carriers, sericin can form hydrogels through physical, chemical, and photo-crosslinking methods, and the most common chemical crosslinking method is to prepare sericin hydrogels with glutaraldehyde as a crosslinking agent [42]. For example, by taking advantage of the fact that proteins do not dissolve in ethanol, pure sericin hydrogel containing no other substances can be prepared via the ethanol precipitation method and the ultrasonic method [43]. Photo-crosslinking can also be employed, as Qi et al. [44] reported when they formed an in situ hydrogel by photo-crosslinking sericin functionalized into methylacryloylsericin under UV irradiation.

Cellulose is a kind of natural polymer compound formed by the dehydration of multiple glucose monosaccharide molecules and glycosidic bonds. It is an important component of the plant cell wall and the most abundant biological material in nature. In addition, cellulose also has the characteristics of high mechanical strength and good chemical stability. Therefore, it is widely used in biomedicine, food chemicals, and other fields. In the actual preparation process, inflammation can be still induced by some other substances that are released from the cellulose-based hydrogels during the process of them contacting with the body, although cellulose itself can be biodegraded by biomolecular interactions in the body [45]. In the field of sustained-release drug carriers, cellulose can form hydrogels through physical and chemical crosslinking. The mechanism of physical crosslinking is as follows: cellulose has many hydroxyl groups, which can be connected into a network structure by forming hydrogen bonds [46]. The mechanisms of chemical crosslinking to form hydrogels can be divided into two categories: crosslinking through chemical crosslinking agents (such as propylene oxide and divinyl sulfone) or covalent crosslinking between cellulose molecules by forming free radicals [47].

Starch is a kind of plant polysaccharide that widely exists in plant seeds, fruits, and roots. It is characterized by its abundant sources, low cost, biodegradability, nontoxicity, and harmlessness, and it has been widely used in many fields [48]. Currently, cassava starch hydrogel is the most studied starch hydrogel and has many advantages such as high hydrophilicity, a high swelling degree, and a three-dimensional structure conducive to drug delivery. In addition, the nano-modified starch also has the advantages of small particle size, nontoxicity, biodegradability, nonimmunogenicity, and histocompatibility; thus, it is a material with great potential for development [49]. Cassava starch can form hydrogels via physical and chemical crosslinking. Although hydrogels prepared using physical methods have the advantages of no environmental pollution and high yield, they also have the disadvantages of high energy consumption in the preparation process and poor structural stability; thus, the application of cassava starch hydrogels prepared by physical methods is poor. Currently, the commonly used cassava starch hydrogel is prepared by modified grafting of cassava starch (using a grafting agent such as acrylamide) [50] (Figure 3d).

### 2.2. Synthetic Hydrogels

NIPAM is a white crystal with a melting point of 60 °C, a boiling point of 90 °C, and a minimum critical phase transition temperature of 32 °C that can be used for the synthesis of heat-sensitive materials and expansive macromolecular hydrogels [51]. In the field of controlled-release drug carriers, NIPAM can form a hydrogel using a double bond, which is an initiator that triggers the NIPAM monomer or crosslinking agent to initiate polymerization by chemical crosslinking (such as free-radical polymerization). This method is relatively simple, but the initiator and crosslinking agent that remain in the hydrogel and other substances may influence the performance of the hydrogel. Physical crosslinking (such as illumination, ultrasound, and radiation) is a way to physically induce the polymerization of the NIPAM monomer to form a hydrogel without the addition of other substances. Hydrogels prepared via this method have attracted much attention this year because there is no interference from other substances [52]. In addition, NIPAM hydrogel not only has the characteristic of biodegradability, but also can generate an effective immune response via the proinflammatory fragments that are produced by acidification degradation in the body; thus, it has a wide range of applications in biomedicine, human tissue materials, and other fields.

**Figure 4:**
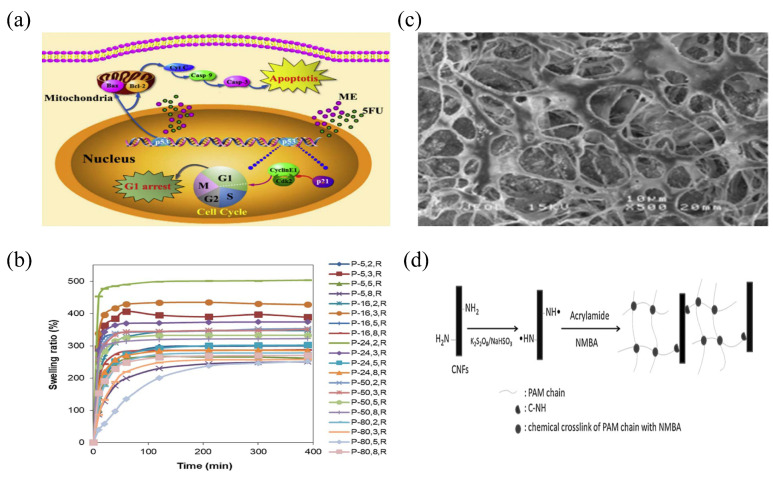
The action mechanism, swelling ratio, preparation flow chart, and internal structure diagram of some synthetic hydrogels. (**a**) The schematic illustration of synergistic antitumor effects of combination treatment of ME and 5FU released from the PFA/PPLL hydrogels. Reprinted with permission from Ref. [53]. Copyright 2016, Wu, X. (**b**) Swelling ratio of the dried PVA hydrogels at room temperature, pH 7.4. Reprinted with permission from Ref. [56]. Copyright 2021, Boran, F. (**c**) SEM image of PEO composite hydrogel. Reprinted with permission from Ref. [60]. Copyright 2019, Kanafi, N.M. (**d**) Schematic diagram of covalent bonding mechanism of PAM composite hydrogel. Reprinted with permission from Ref. [64]. Copyright 2011, Zhou, C.

PEG, a synthetic polymer with advantages of good biocompatibility, low toxicity, and degradability, has been widely used in nerve, cartilage, bladder, and other tissue engineering fields [53] (Figure 4a). As a hapten, when PEG material is used alone, its immunogenicity is very low and usually does not cause an immune response of the body. However, when PEG material is modified to form a conjugate, its immunogenicity will be improved and there is a certain probability of causing the immune response of the body to produce specific antibodies against PEG. In the field of tissue engineering, PEG often plays a role in the formation of hydrogels. The crosslinking methods for preparing hydrogels include high-energy radiation, functional group reactions, and free-radical polymerization. According to their action form, hydrogels prepared via the high-energy radiation method can be classified as physically crosslinked. The advantage of hydrogels prepared using this method is that the crosslinking condition is mild, whereas the disadvantage is that there are unreacted free radicals in the resulting hydrogels. Free-radical polymerization refers to the use of the PEG monomer as the polymerization initiator to form a hydrogel. The disadvantage of this method is that there will be some initiator residue in the hydrogel, which may affect its performance. The functional group reaction method refers to the preparation of a hydrogel by forming covalent crosslinking of the reaction between the terminal functional groups of derivatives of PEG and the terminal functional groups of another derivative of PEG (they are complementary). The advantage of this method is that there is no additive; hence, it is relatively safe [54].

PVA, obtained from the hydrolysis of polyvinyl acetate, is a water-soluble, nontoxic, and nondegradable polymer. It contains a large number of hydroxyl groups in its molecules and has good mechanical properties and biocompatibility. It has been widely used in the field of biomedicine [55]. Pharmacological experiments have proven that PVA is nontoxic, odorless, and nonirritating to the skin and does not cause allergic reactions; therefore, it is widely used in the field of drug carriers. In the field of sustained-release drug carriers, PVA mainly uses hydrogels to exert its effect. PVA can form hydrogels through physical and chemical crosslinking methods, and glutaraldehyde is usually used as the crosslinking agent. This method is simple and has a short cycle. However, the use of glutaraldehyde affects the cell affinity of PVA hydrogels. Physical methods are commonly used to prepare PVA hydrogels via cyclic freeze-thawing technology, which is characterized by its high efficiency, simplicity, and absence of chemical residue [55,56] (Figure 4b).

PEO is a high molecular weight polymer produced by ring-opening polymerization of ethylene oxide with high viscosity, water solubility, low chemical toxicity, good histocompatibility, and immunologic unresponsiveness, which has been widely used in medicine, agriculture, and other fields [57]. In terms of biodegradation, PEO hydrogel with a low relative molecular weight can be biodegraded in the body and its degradation products can be discharged out of the body, but when the relative molecular weight of PEO reaches a certain value, it cannot be biodegraded in the body. In the field of tissue engineering, PEO often plays a role in the formation of hydrogels. Studies have shown that PEO hydrogels can be prepared by physical crosslinking (such as photo-crosslinking and radiation crosslinking) and chemical crosslinking (such as by adding a crosslinking agent) [58,59]. At present, it is a new hot spot in the research of drug carrier hydrogels to prepare composite hydrogels using synthetic polymer materials such as PEO. For example, Norizah et al. [60] prepared composite hydrogels of hydroxylmethyl cellulose and PEO using citric acid as a crosslinking agent and hydroxylmethyl cellulose and PEO as materials. This composite hydrogel had better water coagulation performance than the single material (Figure 4c).

Acrylamide (AM) is a white crystal that is soluble in water, methanol, ethanol, and other polar solvents. AM can easily decompose in alkaline solutions, but it can be stable in acidic solutions. When heated to the melting point or under ultraviolet radiation, AM can easily polymerize. In addition, AM has slight toxicity and can produce neurotoxic effects on various animals to different degrees [61]. Nevertheless, it is still widely used in sewage treatment, the construction industry, the paper industry, bioengineering, and other fields due to its good adhesion, dispersity, and biocompatibility [62]. In the field of tissue engineering, polyNIPAM, a derivative of AM, has been widely studied and can be prepared by physical crosslinking (such as radiation crosslinking) and chemical crosslinking (adding crosslinking agents such as nanostructure particles) to form a single hydrogel or a composite hydrogel prepared by mixing polyNIPAM with other substances [63,64] (Figure 4d).

## 3. Characterization Techniques for Hydrogels

As a material widely used in many fields and with the continuous expansion of the application of hydrogels, the industry is putting forth higher requirements for their performance. In order to meet the application needs of various fields, hydrogels that can be used as sustained-release drug carriers need to be subjected to advanced characterization before application. The present study describes the following characterization methods of hydrogel materials: spectroscopic analysis, scanning electron microscopy (SEM), swelling detection, and differential scanning calorimetry (DSC).

Current spectroscopic analysis methods for hydrogel characterization include Fourier-transform infrared (FTIR) and nuclear magnetic resonance spectroscopy (NMR). The role of spectroscopic analysis in hydrogel characterization is mainly to detect the chemical composition of the hydrogel. Thus, it can be determined whether the molecular chains of hydrogels are connected via chemical bonds by observing the variation of absorption peaks of chemical groups and the deviation of characteristic peaks, allowing the crosslinking mode of the hydrogel prepared to be determined. Arndt et al. [65] characterized the crosslinking mode of a hydrogel prepared by a mixture of polyacrylic acid and polyacrylic alcohol through FTIR, and the research results showed that the crosslinking mode of the mixed hydrogel was chemical crosslinking, which mainly depended on ester bond interactions to form the hydrogel.

SEM, whose main principle is to use an electron beam to penetrate the sample to be tested followed by focused imaging, can be used to detect the fine structure of the sample to be tested. Because of its high resolution and magnification, it is often used to detect the surface morphology and internal fine structure of the hydrogel in the characterization of hydrogel materials. For example, Kim et al. [66] observed methacrylate anhydride–glucose hydrogel samples prepared by light crosslinking after freeze-drying and natural drying by SEM, and the observation results showed that the freeze-drying hydrogel samples had an obvious pore structure, whereas the samples dried in a natural state had a less evident pore structure.

Swelling detection is also one of the common methods for characterization of hydrogel materials. Its purpose is to test the absorption or release ability of hydrogel materials as a sustained-release drug carrier matrix. The specific detection method is to test the swelling rate of hydrogel materials by controlling temperature, pH, or time in a specific solution condition. For example, Khurma et al. [67] measured the expansion curve and swelling rate of a chitosan–PEG semipermeable hydrogel under different pH, temperature, and PEG content through a swelling test experiment.

The principle of DSC is based on evaluating the physical changes in a stable material according to the temperature. DSC is often used to characterize the phase transition temperature of hydrogel materials and the influence of polymers on the phase transition temperature in the characterization of hydrogel materials. For thermosensitive hydrogels, when the ambient temperature reaches the phase transition temperature, water will be separated from the hydrogels, followed by a certain phase transition behavior. This process is accompanied by a certain thermal effect; therefore, the DSC method can be used for detecting the phase transition of thermosensitive hydrogels. For example, Kim et al. [68] used DSC to characterize the existence state of water in a hydrogel prepared with PVA and chitosan, and the experimental results showed that water in the hydrogel could be divided into bonded water and free water.

## 4. Responsive Hydrogels

**Figure 5:**
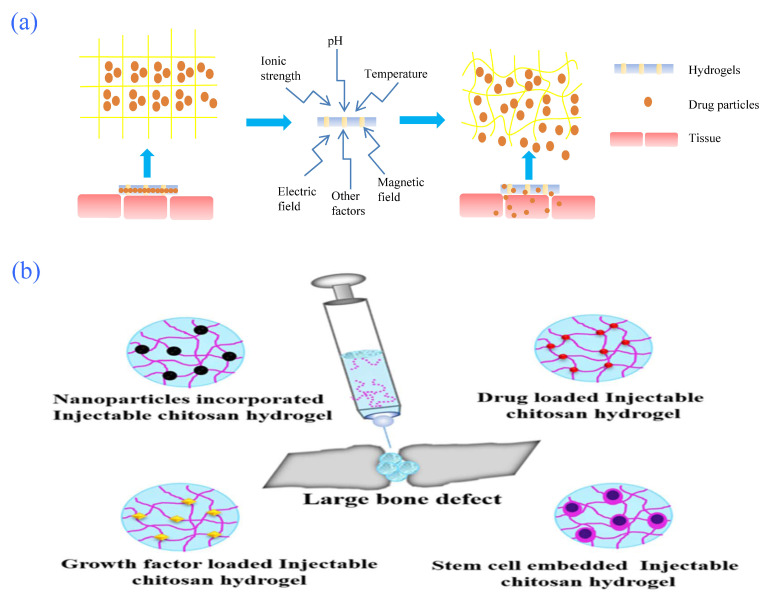
Response mechanism of intelligent hydrogel and its application in promoting bone growth. (**a**) Response process of intelligent hydrogel. (**b**) Thermo/pH-responsive CS hydrogels as delivery carriers for bioactive molecules, NPs, drugs, and cells at the defective site, enhancing bone growth. Reprinted with permission from Ref. [72]. Copyright 2020, Lavanya, K.

A hydrogel is a kind of three-dimensional network polymer that contains hydrophilic groups in its interior and does not dissolve when swollen by water. Hydrogels can be divided into traditional hydrogels (insensitive to external stimuli) and sensitive hydrogels (sensitive to external stimuli) according to their different responses to external stimuli; the latter are also known as intelligent or smart hydrogels [69,70]. External environmental stimulation includes temperature, pH value, temperature-pH, ionic strength, organic compound concentration, magnetic fields, electric fields, and light. When these external factors change to a certain critical point, smart hydrogels usually undergo a discontinuous sudden change or volume phase transformation [71]. Intelligent hydrogels have not only high biocompatibility and flexible synthesis methods but also the advantages of high stability and few side-effects; therefore, they have been widely used in the field of clinical treatment. They can not only transport drugs for targeted release (Figure 5a) to achieve the purpose of targeted therapy, but also embed cells and serve as scaffolds for tissue repair.

### 4.1. pH-Responsive Hydrogels

In external stimulation, pH is widely used; thus, one of the most widely researched intelligent-responsive hydrogels is the pH-responsive hydrogel. Generally, this type of hydrogel contains pH-sensitive acidic and basic groups, including carboxyl and amino groups, or pH-sensitive dynamic covalent bonds, relying on these groups or chemical bonds to achieve controlled drug release [72] (Figure 5b). Under pathological conditions, the pH of body tissues changes, leading to ionization of acidic and basic groups capable of dissociation, hydrogen bond dissociation between macromolecular chains in the gel network [73], and electrostatic interaction, ultimately shrinking or swelling the hydrogel to achieve the purpose of controlled drug release. The second condition that leads to the degradation of hydrogels is the dissociation of dynamic covalent bonds in the hydrogel system, thereby controlling drug release. Hydrogels for the controlled release of drugs in this manner are often used for the treatment of body parts with obvious pH differences, such as the gastrointestinal tract [74], as well as for cases where pH values in pathological states, such as bacterial infections and cancer cell lesions, differ greatly from the normal state [75].

pH-responsive hydrogels can be divided into anionic, cationic, and amphoteric pH-responsive hydrogels, according to the different groups in the hydrogel that respond to pH [76]. The anions contained in the anionic photo-responsive hydrogel network are usually carboxyl groups [77], and the presence of carboxyl groups reduces the dissociation degree of ionizable groups. At this time, hydrogen bonding does not occur between the carboxyl group and water molecule, but is largely concentrated between the polar groups of large molecules; thus, water molecules do not easily enter the hydrogel, and the hydrogel shrinks. Cationic photo-responsive hydrogels usually contain amino groups or amino groups replaced by hydrocarbon groups. Protonation or deprotonation of amino groups can occur in different pH environments. When in an alkaline environment, the presence of amino groups reduces the degree of dissociation of ionizable groups, and hydrogen bonding is mainly manifested as hydrogen bonding between amino groups rather than between amino groups and water molecules. In this case, water molecules do not easily enter the hydrogel, and the swelling of the hydrogel is reversed. Zwitterionic photo-responsive hydrogels contain both acidic groups (such as carboxyl groups) and basic groups (such as amino groups), and the comprehensive application of zwitterionic group ionization makes hydrogels responsive to pH.

### 4.2. Temperature-Responsive Hydrogels

Temperature-responsive hydrogels control drug release according to changes in the ambient temperature. These kinds of hydrogel networks usually contain polymers with a “dissolved-insoluble” phase transition within a certain temperature range. This phase transition is usually divided into two situations. The first situation has the lowest critical solution phase transition temperature (LCST) [78,79,80,81]. The main and side-chains of polymers in such hydrogels usually contain hydrophobic groups, such as methyl, ethyl, and propyl groups, as well as some hydrophilic groups, including ether bonds or amide bonds. When the temperature rises to the critical temperature, phase separation occurs in thermosensitive polymers (i.e., the transformation from the dissolved state to the insoluble state). The second type has the highest critical solution phase transition temperature (UCST). The main and side-chains of the polymers in this type of hydrogel usually contain associative polar groups dominated by amphoteric polyelectrolytes [82,83]. When the temperature rises to the critical temperature, the polymer undergoes phase separation (i.e., transformation from an insoluble state to a dissolved state).

At present, the developed temperature-responsive hydrogels that respond to human body temperature changes are mainly divided into three categories: positive thermal-sensitive type, negative thermal-sensitive type, and heat-reversible type. Thermosensitive hydrogels have the highest critical solution phase transition temperature, and the main force at low temperature is intramolecular electrostatic or hydrogen bonding in hydrogels. Hydrogen bonding between hydrophilic groups in the hydrogels and water molecules is weak, whereby water molecules do not easily enter the hydrogens, and the hydrogels shrink [84]. Negative thermosensitive hydrogels have the lowest critical solution phase transition temperature, whereby the hydrogen bonds between hydrophilic groups in hydrogels and water molecules are stronger; thus, water molecules are more likely to enter the hydrogels, and the hydrogels swell. Thermally reversible hydrogels are mainly prepared by physical crosslinking methods, such as van der Waals forces and hydrogen bonding. Phase transformation can be achieved under certain conditions, and the volume usually does not change [76,85].

### 4.3. Electric Field-Responsive Hydrogels

**Figure 6:**
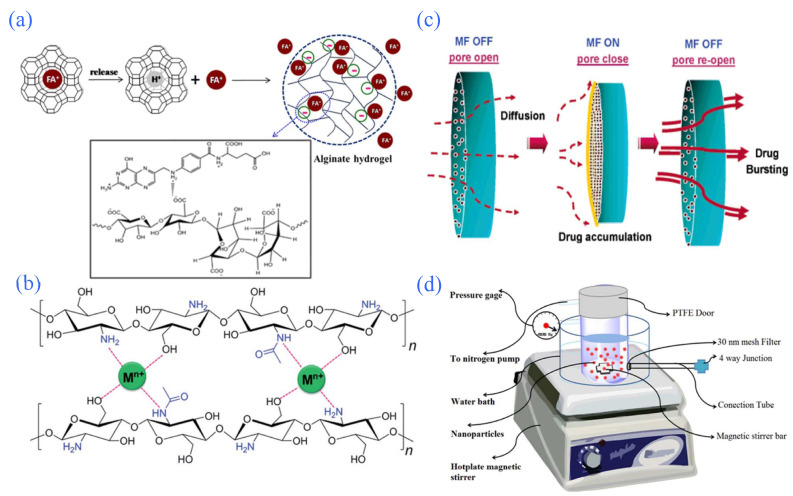
Partial intelligent-response hydrogel controlled-release system. (**a**) Proposed mechanism of FA release from zeolite FAY/alginate hydrogel. Reprinted with permission from Ref. [88]. Copyright 2015, Paradee, N. (**b**) Chemical structures of chitosan and their interwoven networks driven by the complexation between metal ions and OH and NH_2_ groups in the chitosan chains. Reprinted with permission from Ref. [95]. Copyright 2015, Sun, Z. (**c**) Mechanism of “close” configuration of the ferrogels due to the aggregation of Fe_3_O_4_ nanoparticles under a magnetic field causing the porosity of the ferrogels to decrease. Reprinted with permission from Ref. [97]. Copyright 2006, Liu, T. (**d**) Schematic of proposed device used for measuring the drug release from nanoparticles under pressure. Reprinted with permission from Ref. [106]. Copyright 2017, Hosseinifar, T.

Electric field-sensitive hydrogels (EFSHs) have ionized groups (such as sulfonic groups, amide groups, and sulfa groups) in the network, which is an important condition for polymer materials to have electrical stimulation-responsive behavior [86]. Electric field-sensitive hydrogels are usually composed of polyelectrolytes. When stimulated by an electric field, the gel in the electrolyte solution changes in shape or volume, which mainly includes swelling elimination, as well as swelling and bending deformation of the gel, thus realizing the conversion of electric energy to mechanical energy. Positive groups in the hydrogel generate water at the anode, and negatively charged groups generate water at the cathode [87], resulting in changes in the internal and external osmotic pressures of the hydrogel due to differences in internal and external ion concentrations and ultimately leading to morphology changes and achieving the purpose of drug release control [88] (Figure 6a). At the same time, because of the various deformation characteristics of electric field-responsive hydrogels in electric fields, they have broad application prospects in artificial muscles and controlled drug release, including as bionic actuators, artificial muscles, and chemical valves [71]. Regarding bionic actuators, Nagata et al. [89] developed an artificial reptile in 1992, which was the first successful experiment to achieve flexible movements of animals using gel as a material. The artificial reptile was made of PAMPS electrolyte gel soaked in a salt solution containing surfactants. When the solution was energized, the gel could stretch, bend, and move forward. Regarding artificial muscle, Hamlen et al. [90] proposed in 1965 that electric field-responsive hydrogels could be developed into artificial muscle. They found that the gel fibers made of PAANa and PVA shrank and swelled in response to direct current for several minutes. Later, Moschou et al. [91] added a mixture of conductive properties to PAA/PAM hydrogels and developed a new type of artificial muscle material, which has the advantages of fast electric braking and electric responsiveness in medium-and small-voltage environments in neutral solutions.

### 4.4. Ion-Responsive Hydrogels

When ion-sensitive hydrogels are under the condition of low ion concentration, counter ions have difficulty entering the gel and the ionization degree of ionizable groups is low. The ionization degree increases when the ion concentration increases, the gel swelling increases, and then the gel ionization degree reaches the maximum. At this time, the ionic osmotic pressure between the gel and the solution decreases, and the gel swelling decreases [92]. Many metal ions play important roles in the human body, such as regulating protein function and nerve conduction, maintaining acid-base balance in the internal environment, mediating muscle contraction, functioning as enzyme active components, and participating in the regulation of intracellular osmotic pressure. These activities involve the macro metal elements Na^+^, K^+^, Ca^2+^, and Mg^2+^, the trace elements Fe^2+^, Zn^2+^, etc., and the ultramicro metal elements Mn^2+^, Cr^3+^, etc. In different body fluids, the types and contents of metal ions are also different, and each organ also has a specific ionic strength [93,94]. In today’s society, there are many heavy-metal pollution problems, such as excessive heavy metals in water leading to excessive heavy metals in rice and metal pollution throughout the environment, which results in the enrichment of heavy-metal ions in the food chain and causes a major public security problem. Sun et al. [95] developed a new multistimulus-responsive hydrogel in 2015 (Figure 6b). By mixing chitosan with multiple metal ions in an appropriate pH environment, a series of transparent and stable hydrogel complexations through supramolecular interactions could be observed in a very short time (most of them occurred in approximately 2 s). If other high-valence metal ions are introduced into a supramolecular gel, the original complexation of metal ions on chitosan will be broken, and the hydrogel will be converted into a sol.

### 4.5. Magnetic Field-Responsive Hydrogels

The volume of magnetic field-sensitive hydrogels can expand and contract under the action of a magnetic field. Such materials usually contain inorganic magnetic nanoparticles in the gel, and the nanoparticles can be fixed in a three-dimensional crosslinked network of the hydrogel by chemical bonding or physical embedding methods. When affected by the magnetic field effect, magnetic particles immediately gather, the hydrogel network contracts, and the solvent is “crowded out”, making the hydrogel shape change rapidly [96,97] (Figure 6c). A large number of scholars have shown that almost all organisms contain magnetic ions, such as Fe^2+^ in hemoglobin and Cu^2+^ in hemocyanin, which can respond to an external magnetic field. Therefore, magnetic field-responsive hydrogel controlled-release drug systems have attracted great attention in the academic world. The preparation of magnetic field-responsive hydrogel controlled-release drug systems is usually based on temperature-responsive hydrogels, on which a variety of magnetic materials containing iron metal oxides, such as Fe_3_O_4_, Fe_2_O_3_, and other ferrite materials, are embedded. When a magnetic field is added to the external environment, drugs will move in the body, oriented under the guidance of the environmental magnetic field, and, when the magnetic field-responsive hydrogels loaded with concentrated drugs are fixed to a body tissue that is in a pathological state, the drugs will be released to achieve targeted controlled release [98]. Under the action of an external magnetic field, these magnetic materials can realize the conversion of energy from magnetic field energy to internal energy such that the temperature of the controlled-release system of the magnetic field-responsive hydrogel drug increases, causing swelling and a volume change, which may also realize the transformation of the “sol-gel” phase. Hawkins et al. [99] embedded magnetic materials and a drug model, lysozyme, in a temperature-responsive hydrogel controlled-release system to prepare a magnetic field-responsive hydrogel controlled-release system. An alternating magnetic field is applied outside the body to produce a thermal effect of the material by using internal energy transformed from magnetic field energy; then, the hydrogel controlled-release drug system responds to the temperature in a sol state, and the lysozyme is released. When the external magnetic field supply is stopped, the thermal effect of the material also disappears and the controlled release system of hydrogel drugs presents a “sol-gel” phase transformation, thus preventing the continued release of drugs and achieving the purpose of controlled drug release.

### 4.6. Pressure-Responsive Hydrogels

Pressure-responsive hydrogel volume phase transformation occurs when the external environmental pressure changes [100,101]. The pressure-response property of the hydrogel was initially obtained through theoretical calculation, which is usually manifested as hydrogel contraction at low pressure and hydrogel swelling at high pressure [102], and usually accompanied by temperature sensitivity. When the temperature is constant, the LCST of the hydrogel increases with increasing pressure, presenting a state of swelling [103]. Pressure-sensitive hydrogels, as drug carriers, can act on tissues with dynamic mechanical environments, such as bone, blood vessels, and muscle [104,105]. Hosseinifar et al. [106] (Figure 6d) prepared a pressure-responsive nanohydrogel using modified β-cyclodextrin and alginate to deliver drugs as a controlled-release system, and they selected 5-fluorouracil as a hydrophobic drug model. The model carrying drugs using this kind of hydrogel can release the drugs under the condition of blood pressure stimulation. In addition, the controlled-release drug systems can be compatible with various cells, such as small-volume colon cancer cells, and they have many other advantages. Pressure-responsive hydrogels have wide application prospects, but research on stress responsiveness is less common.

### 4.7. Photo-Responsive Hydrogels

**Figure 7:**
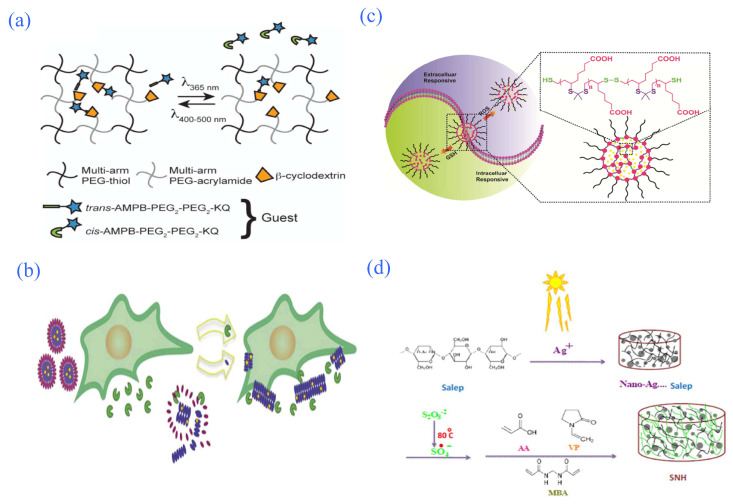
Partial intelligent-response hydrogel controlled-release system. (**a**) Schematic of photoinitiated release of an azobenzene functionalized drug from a β-cyclodextrin-containing PEG-based reservoir. Reprinted with permission from Ref. [108]. Copyright 2016, Nehls, E.M. (**b**) Schematic diagram of the killing effect of enzyme-responsive injectable hydrogels on cancer cells. Reprinted with permission from Ref. [113]. Copyright 2016, Kalafatovic, D. (**c**) Redox-responsive controlled-release system based on TKN hydrogel. Reprinted with permission from Ref. [114]. Copyright 2018, Chen, D. (**d**) Proposed mechanism for preparation of SNH. Reprinted with permission from Ref. [116]. Copyright 2016, Hooshyar, Z.

The size of a photosensitive hydrogel can change due to radiation, as its interior contains light-sensitive groups. These groups undergo light fracture, isomerization, and light dimer formation under different light conditions. The dipole moment and group conformation changes make the gel swell and shrink, moving between a colloidal state and a solution state. When the volume of the hydrosol changes due to the light response, the structure of the controlled polymer network may change, releasing the substances loaded in it. The current research shows that, in light-responsive hydrogels, the controlled-release mechanism can be divided into two kinds: photochemical mechanisms and photothermal mechanisms. In the photochemical mechanism, photosensitive groups in sustained-release systems undergo a chemical reaction of polymerization and isomerization through an effect of light, causing changes in macromolecular chain conformation, dipole moments, electrical conductivity, or ion concentrations in the slow-release system. Then, the swelling volume of the hydrogel changes, and drug release is achieved. In the photothermal mechanism, when there is light irradiation, the photosensitive groups in the hydrogel are converted into heat energy under the action of light, which increases the local temperature of the hydrogel. When the temperature reaches the phase transformation temperature of the hydrogel, the hydrogel undergoes phase transformation to achieve controlled drug release. According to different response wavelengths, photo-responsive hydrogels can be further divided into visible, ultraviolet, and infrared photo-responsive hydrogels [107,108,109] (Figure 7a).

### 4.8. Biomolecule-Responsive Hydrogels

Biomolecule-responsive hydrogels refer to hydrogels that can have corresponding responses to specific molecules of organisms (such as glucose, enzymes, and DNA). Currently, bio-responsive hydrogels are widely studied, including glucose-responsive hydrogels and enzyme-responsive hydrogels. The glucose response of the hydrogel preparation mechanism mainly occurs through the presence of glucose oxidase (GOD) in pH-responsive hydrogels. Its action mechanism involves converting glucose molecules into gluconic acid using a hydrogel containing GOD and changing the pH of the environment where hydrogels are used to achieve the release of drug particles by using the sensitivity of pH hydrogels to environmental acid-base changes. Such hydrogels are sensitive to changes in blood glucose concentration in the body and have broad prospects in the treatment of diabetes and diseases related to blood glucose [72,110,111]. The mechanism of action of enzyme-responsive hydrogels involves breaking polypeptides and their derivatives which can be cleaved by specific enzymes. When these enzymes are introduced into the hydrogel, failure of the hydrogel structure occurs. The current specific enzymes that can be used to destroy hydrogels mainly include glutamic aminotransferase, protease, lysine oxidase, and esterase [112,113] (Figure 7b). For example, Kalafatovic et al. added the amphiphilic peptide GFFLGL-DD to a breast cancer cell line and the cell matrix released metalloproteinase MMP-9, catalyzed the amphiphilic peptide to break and release the self-assembly unit, transformed the self-assembly morphology from spherical micelles to fibers, and released the antitumor drug doxorubicin [114].

### 4.9. Redox-Responsive Hydrogels

The theoretical basis for the design of redox hydrogels is that in-depth research on the difference in redox potential between tissues of cancer lesions and normal tissues in recent years has found that, in normal tissues, due to the reduction in NADPH and glutathione reductase, the concentration of reduced glutathione (GSH) in the cell solute and nucleus can reach 10 mmol/L, whereas the concentration of extracellular GSH is only 2–20 μmol/L. However, the concentration of GSH in the cells of cancerous lesions is approximately four times that of normal tissue cells; hence, they are in a reduced state, whereas outside of the cancer cells there is an excess of reactive oxygen species, which produces an oxidized state. Due to these differences, scientists have been prompted to explore how to take advantage of this condition. A drug can be designed that can target therapy according to the difference in the redox state of cells. Currently, disulfide bonds are the main groups used in the study of drug sustained release according to the differences in redox states of cells, as disulfide bonds can exist stably in an oxidizing environment, whereas disulfide bonds break and are reduced to sulfhydryl groups (–SH) when in a reducing environment. For example, Chen et al. [114] prepared an oxidation-reduced prototype hydrogel that can respond to both ROS and GSH using the antioxidant lipoic acid (LA) to synthesize ketal oligomers through the disulfide bonds formed through sulfhydryl groups (–SH) at both ends of the ketal oligomers (Figure 7c).

### 4.10. Multi-Responsive Hydrogels

A single response of a hydrogel controlled-release drug carrier often cannot achieve the ideal treatment effect because of the complexity of the human physiological environment and the diversity of the lesion site environment; thus, scientists have begun to study a new kind of intelligent hydrogel in which several response performances can converge on the same carrier. The gel can choose the right means of response according to the characteristics of the environment to achieve the ideal effect, which has huge development prospects [115,116] (Figure 7d). At present, scientists have developed hydrogel slow-release systems with double and triple corresponding properties. For example, Chen et al. [117] prepared pH/temperature dual-responsive hydrogel controlled-release drug systems using UV light-induced poly(*N*-isopropylacrylamide), carboxymethyl chitosan (CMCT), and arginine branch crosslinking. Mahdavini et al. [118] introduced magnetite nanoparticles into a hydrogel prepared from carrageenan and sodium alginate and developed a magnetic/pH dual-responsive hydrogel controlled-release drug system. Wang et al. [119] prepared a core-shell complex hybrid magnetic field/near-infrared dual-responsive hydrogel controlled-release drug system using poly (*N*-isopropylacrylamide-acrylamide) with a thermal response, fluorescent carbon points embedded in porous carbon shells, and superparamagnetic iron clustered in the core center. In addition, Eskandari et al. [120] developed a triple-responsive hydrogel controlled-release drug system with pH/temperature/ion-responsive properties that could regulate drug release by adjusting pH, temperature, and ion strength when loaded with drugs to achieve the best clinical effect.

## 5. Application of Hydrogels in Tissue Engineering

Because of their unique structure, hydrogels indirectly determine their good performance. For example, gel prepolymers in solution can uniformly wrap cells or drugs, thus realizing the loading and transportation of cells or drugs. In addition, the water environment inside the hydrogel can accurately mimic the three-dimensional microenvironment of tissue cells. Lastly, some hydrogels are injectable and can achieve in situ regeneration and reorganization of tissues. Because of these excellent properties, hydrogels are widely used in tissue engineering fields such as skin trauma, bone defects, arthritis, cartilage defects, and tumor treatment [121].

### 5.1. Skin Trauma

**Figure 8:**
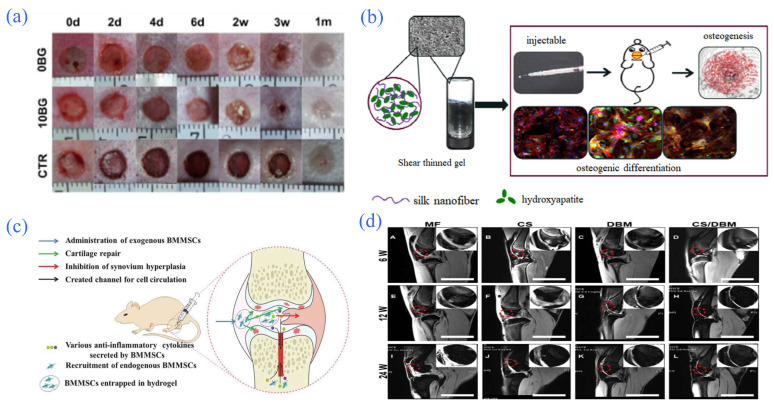
Application of hydrogel in partial tissue engineering. (**a**) Process of wound healing promoted by the hydrogel. Reprinted with permission from Ref. [125]. Copyright 2015, Zeng, Q. (**b**) Involvement of hydrogels in repairing bone defects. Reprinted with permission from Ref. [128]. Copyright 2017, Ding, Z. (**c**) Schematic diagram of the possible mechanism of hydrogels loaded with bone marrow mesenchymal stem cells in alleviating collagen-induced arthritis. Reprinted with permission from Ref. [135]. Copyright 2015, Liu, H. (**d**) Specimen of rabbit cartilage after allogeneic chondrocyte transplantation and repair of demineralized bone matrix composite scaffold using chitosan hydrogel. Reprinted with permission from Ref. [139]. Copyright 2016, Man, Z.

Skin trauma refers to the structural damage of skin tissue caused by various external factors, which can be divided into bruising, stabbing, laceration, and slashing. The common symptoms of trauma are bleeding, oozing of blood, epidermal shedding, etc., whereas certain inflammatory reactions and infections generally occur during the repair process [122]. Skin injury is generally treated through the body’s self-repair mechanism, but when the skin wound exceeds a certain level and the regenerative epidermis cannot cover the wound, skin grafting is used for treatment [123]. Current research shows that, when hydrogels are used as skin wound dressings, they can not only effectively prevent the infection of exogenous microorganisms but also prevent the loss of wound fluid and provide a moist microenvironment for wound healing. Hydrogels also have good oxygen permeability, which is conducive to wound healing [124]. Although hydrogels as wound dressings have played positive roles in treating injuries, the load of a bioactive hydrogel can further promote the healing of wounds. As reported by Zeng et al., a thermosensitive hydrogel was prepared by combining agarose with SA and bioclasts. This hydrogel could be gelated by the interaction between agarose and SA at approximately 37 °C, and the bioglass could make the previously formed secondary crosslinked gel by releasing ions itself. Studies have shown that hydrogels can promote the proliferation and migration of fibroblasts and endothelial cells, thus improving the ability of blood vessel formation and promoting wound healing [125] (Figure 8a).

### 5.2. Bone Defects

Bone defects refer to the structural incompleteness of bone tissue caused by trauma, infection, congenital malformation, tumor, etc. When bone defects reach a certain range, it is difficult for the body to heal by itself. At present, the commonly used treatments for bone defects are self-healing or bone defect material implantation to promote bone repair [126]. In the treatment of bone defects, hydrogels are mainly made into scaffolds or injectable gels, and their excellent histocompatibility is used to promote cell adhesion to repair bone defects. For example, Ding et al. [127] used SF nanofibers as the main material, as well as hydrophobic hydroxyapatite particles imitating the extracellular matrix of bone, to prepare a composite scaffold of SF and hydroxyapatite, and they loaded it with controlled bone morphogenetic protein-2 (BMP-2) to promote bone formation. The microenvironment of the bone defect was optimized successfully. The team also used an injectable nano SF/irregular hydroxyl apatite compound hydrogel to repair bone defects in subsequent experiments. The experimental results showed that new bone tissue and bone defect healing were detected in the model rats, and the repair effect of composite hydrogel was better than that of the single nano SF hydrogel [128] (Figure 8b). Zhao et al. [129] studied embedded human umbilical cord mesenchymal stem cells in alginate saline gel microspheres and mixed such microspheres with a calcium phosphate/chitosan/fiber composite paste to prepare a new composite material for bone defects. Studies showed that the mechanical properties of the composite material could match the cancellous bone of the body. At the same time, it could maintain the vitality of human umbilical cord mesenchymal stem cells and promote osteogenic differentiation. Kim et al. [130] added polysulfonates that could mimic heparin to chitosan hydrogels so that BMP-2 could maintain stable biological activity. In this hydrogel, bone marrow mesenchymal stem cells (BMSCs) showed a tendency to differentiate into the osteogenesis pathway.

### 5.3. Arthritis

Arthritis refers to inflammatory changes in the joints and surrounding tissues caused by infection, trauma, inflammation, or other factors. Clinical manifestations include joint redness, swelling, pain, and dysfunction. Clinically, arthritis can be roughly divided into rheumatoid arthritis, gout arthritis, osteoarthritis, and infection-related arthritis [131]. The current treatment methods for arthritis diseases mainly include drug therapy, surgical therapy, new drug-targeting therapy, and emerging nanodrug delivery therapy [132]. Hydrogels with good biocompatibility play a very important role in drug treatment; for example, the polyethylene glycol–polylactic acid–glycolic acid copolymer can be used as a gel matrix to carry triamcinolone acetonide, which is commonly used to treat rheumatoid arthritis. A study confirmed the obvious advantages, including the gel system’s stable quality, durability, ability to effectively inhibit inflammation, and slow-release effect [133]. PEG–poly(lactic acid)-glycolic acid (PLGA) can also be used as a carrier for the treatment of rheumatoid arthritis via intra-articular injection. This sustained-release drug delivery system has the advantages of high drug utilization and few side-effects [134]. Scholars such as Liu et al. [135] also attached bone marrow mesenchymal stem cells to a hydrogel material for the treatment of rheumatoid arthritis, and they used fiber gel and hydrogel as carriers of bone marrow mesenchymal stem cells for the treatment of rheumatoid arthritis, achieving good therapeutic effects in an experimental model (Figure 8c).

### 5.4. Cartilage Defects

Cartilage defects are caused by inflammation and degeneration due to the trauma of irreversible cartilage injury and represent a clinically common bone disease. Because there are no blood vessels, innervation, and lymphatic reflux in cartilage tissue, the cell composition of cartilage tissue is relatively singular; thus, once cartilage injury occurs, it is difficult to fully repair by regeneration. Symptomatic osteochondral defects of the current treatment methods include bone marrow stimulation, osteochondral transplantation, chondrocyte transplantation, and cartilage tissue engineering repair, which have emerged in recent years [136]. Natural and synthetic polymers and composite materials play important roles in cartilage tissue engineering repair. Since these materials have good biocompatibility, they can be used as carriers of chondrocytes, which can be inoculated into hydrogels made of natural polymer materials and then transplanted into cartilage defects after the formation of tissue-engineered cartilage [137]. For example, Chen et al. [138] embedded rabbit chondrocytes in glycol hydrogel scaffolds for the repair of rabbit knee cartilage defects. Studies showed that scaffolds could maintain the morphology of chondrocytes in vitro and cause the cells to proliferate for more than 21 days. The hydrogel could provide attachment for the proliferation of carried chondrocytes in vivo; the expression of type II collagen in the cartilage defect was high, and the cartilage defect was significantly improved. In addition, the scaffold can also be made of these materials and loaded with chondrocytes carrying cartilage cells to repair cartilage defects. For example, Man et al. [139] used mineralization of a bone matrix-chitosan hydrogel scaffold carrying cartilage cells to repair a rabbit cartilage injury. Twenty-four weeks after surgery, the stent group had no obvious inflammation, and the cartilage defect was repaired successfully. The repair effect was significantly better than that of the control group (Figure 8d).

### 5.5. Corneal Injury

**Figure 9:**
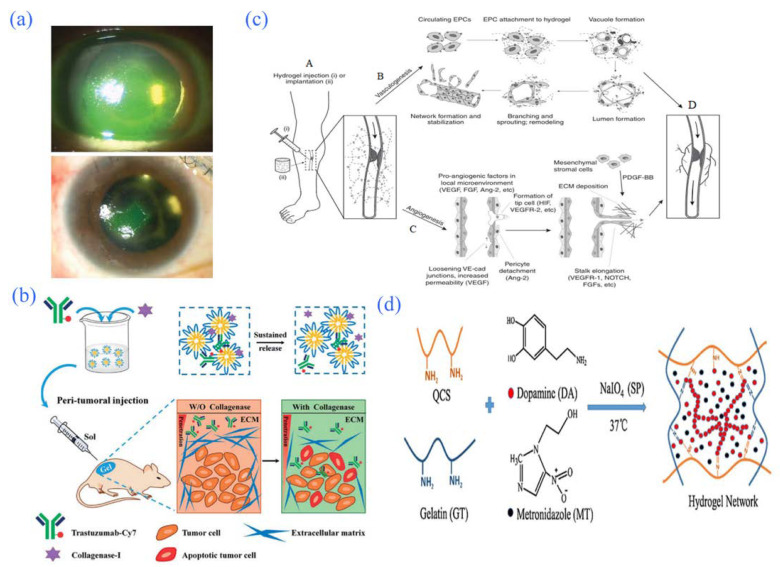
Application of hydrogel in partial tissue engineering. (**a**) Effect of water silica gel hydrogel lens combined with autologous serum eye drops after 4 days of treatment. Reprinted with permission from Ref. [143]. Copyright 2011, Choi, J. (**b**) The antitumor procedures of Col/Tra/Gel. Reprinted with permission from Ref. [150]. Copyright 2018, Pan, A. (**c**) Hydrogel involved in the process of repairing blood vessels. Reprinted with permission from Ref. [152]. Copyright 2015, Blatchley, M.R. (**d**) Schematic diagram of the preparation of chitosan, gelatin, and dopamine sustained-release systems. Reprinted with permission from Ref. [160]. Copyright 2017, Ren, Y.

Corneal injury refers to perforated or nonperforated corneal injuries caused by trauma, corrosion, heat injury, etc. The repair of corneal injuries is a very complex process involving cytokine regulation between epithelial cells, corneal nerves, lacrimal glands, stromal corneal cells, immune cells, and other cells [140]. Current treatments for corneal defects include anti-inflammatory agents, lamellar corneal transplantation, corneal healing promotion, and amniotic membrane transplantation [141]. Hydrogels as sustained-release carriers of therapeutic drugs have great development prospects. For example, Colter et al. [142] developed a new HA hydrogel that can transport loaded drugs to the ocular surface and place them in the lower dome of the eye. Therefore, the hydrogel can be used sustainably for ophthalmic diseases such as local corneal injury. In addition, research shows that SF hydrogels loaded with a UV-crosslinked retinoid can promote the growth and differentiation of human corneal stem cells. These hydrogels also feature high transparency, good elasticity, and tight contact with the cornea; therefore, they are expected to be used as artificial corneas for defect treatment. The hydrogels can also promote the growth and differentiation of human corneal stem cells and the formation of human corneal cells in vitro [38]. Serum is a noncell component that is a part of the fluid in the blood, which is rich in many kinds of growth factors and vitamins. Serum is currently used for the treatment of ocular surface diseases. For example, Choi [143] described a silicone hydrogel lens using autologous serum for the treatment of persistent corneal epithelial defects, and the results showed that the hydrogel lens had a remarkable therapeutic effect (Figure 9a).

### 5.6. Cancer Treatment

Tumors are cells whose genetic material changes and whose growth is out of control under the influence of the external environment (due to chemical, physical, biological, and other factors). According to their degree of harm, tumors can be divided into benign and malignant tumors. Benign tumors are small, but malignant tumors can invade surrounding normal tissue. In severe cases, they can follow the body’s lymphatic system, via which they can be transferred to other body parts and cause serious harm [144]. Therefore, researchers have been searching for treatments that are more effective and produce fewer side-effects to the body, as well as more targeted effects. Among them, intelligent hydrogels have attracted increasing attention in the diagnosis and treatment of cancer. Smart hydrogels can increase the permeability of therapeutic drugs in cancerous tissues, breaking the bottleneck of low permeability of hydrophobic drug delivery [145,146]. Gariepy et al. [147] achieved remarkable results in inhibiting the growth of emT-6 cancer cells in mice by using a chitosan smart hydrogel as the drug carrier in a study in which the therapeutic drug paclitaxel was transported to the tumor site for targeted therapy. Castro et al. [148] injected cisplatin/epinephrine gel (CDDP/EPI/GEL) into tumors for the treatment of recurrent and metastatic squamous cell carcinoma of the head and neck and achieved remarkable results. In previous work, chitosan interventional radionuclide ^188^Re gel was used for the treatment of cancer in mice by locally injecting it into the focal site to prolong the drug’s retention time, which significantly enhanced the antitumor effect [149]. Pan et al. [150] used the PLGA–PEG–PLGA polymer as a gel matrix and added trastuzumab and collagenase to prepare a thermosensitive hydrogel sustained-release system. In experiments evaluating peripheral administration to tumors, the hydrogel systems had little toxicity and could stably exist around tumors for more than 20 days. They also had good tumor cell-killing ability, showing that the hydrogel system had an excellent treatment effect. Moreover, the gel sustained-release system had a better effect after one release compared to the effect when administered four times intravenously. The experiment also strongly confirmed that hydrogels have broad prospects as drug carriers for the treatment of tumors (Figure 9b).

### 5.7. Cardiovascular System

In the field of vascular tissue engineering, there are various materials available on the market for the treatment of cardiovascular diseases, but most of them have the disadvantages of toxic side-effects or low biodegradability; however, hydrogels have great development potential in vascular regeneration. The application of hydrogels in this field is mainly to regulate bone defect repair and angiogenesis by promoting vascularization and changing the performance of hydrogels together with inducing factors [151]. Hydrogels are usually transplanted or injected into sites of vascular injury. There are two mechanisms of angiogenesis using hydrogels. The first is to introduce inducible factors into hydrogels and then make the hydrogels release inducible factors upon the vascular injury to induce angiogenesis. The second involves adhesion of the hydrogel surface to circulating endothelial progenitor cells (EPCs) to promote angiogenesis [152] (Figure 9c). Li et al. [153] developed a controllable, injectable gelatin hydrogel synthesis method for delivering growth factor-induced angiogenesis. Heparin was covalently linked to gelatin, and VEGF was ultimately introduced into gelatin hydrogels by combining the vascular endothelial growth factor (VEGF) with heparin. Then, experiments were carried out on mice. The results show that a gelatin derivative/VEGF is an excellent injection model for a delivery system for soft-tissue regeneration-induced angiogenesis. The experimental results further showed that the material can solve problems related to growth factor deficiency by releasing VEGF on its own. In the treatment of myocardial infarction, hydrogels have also shown great prospects. Injectable hydrogels have currently entered the stage of clinical trials in the treatment of myocardial infarction. The biological mechanisms of its treatment may include the following: promoting angiogenesis, improving blood perfusion in the infarct area and reducing myocardial necrosis, promoting the homing of stem cells and repairing the heart muscle, replacing the extracellular matrix of the myocardium, improving the local microenvironment, and inhibiting the expansion of the infarct area [154].

### 5.8. Nervous System

The nervous system is the most important regulatory system in the human body, as it plays a leading role in regulating and controlling various life systems. With changes in the social environment, neurological diseases such as insomnia, Alzheimer’s disease, and Parkinson’s disease pose a serious threat to human health [155]. As the ability of the nervous system to repair or regenerate traumatic injury is very limited, nerve injury caused by various factors is a major problem in the medical field [156]. Currently, the treatment methods for nerve injury mainly include surgical treatment, gene therapy, tissue engineering, and drug therapy. Tissue engineering treatment for nerve injury mainly involves constructing corresponding tissues in vitro by combining the seed cells and scaffold materials of relevant nerves to support the repair and regeneration of corresponding nerve tissues [121]. A large number of studies have shown that hydrogels are a nerve repair material with great potential. For example, Hopkins et al. [157] prepared hydrogels using SF, fibrin, and collagen as materials and used them as carriers to culture chick embryonic dorsal root ganglia (cDRG). The experimental results showed that SF had a better performance in promoting cDRG growth than fibrin and collagen. In addition, Sun et al. [158] inoculated mouse embryonic stem cells with a SF–gelatin composite hydrogel in different proportions to explore its effect on neural differentiation in mouse embryonic stem cells. The results showed that the composite hydrogel changed the differentiation fate of embryonic stem cells compared with the control group of cell culture plates. As an example, Parkinson’s disease is a common neurodegenerative disease in the elderly, and dopamine is a drug that can be used for Parkinson’s treatment [159]. Ren et al. [160] prepared an injectable hydrogel based on chitosan and gelatin, in which dopamine and metronidazole drugs were embedded. In the experiment, the hydrogel showed stable mechanical properties, good biocompatibility, and biodegradability, and it demonstrated its utility as a sustained-release drug system that can be used for Parkinson’s treatment (Figure 9d).

### 5.9. Reproductive System

Diseases of the reproductive system refer to male reproductive system disease, female reproductive organ dysplasia, and malformation, inflammation, and tumor formation in the reproductive organs. These diseases can be divided by sex classification into male and female reproductive diseases. Diseases of the male reproductive system include wrapping that is too long, foreskin and penile adhesions, testicular damage, biological surface increase in the epididymis, and testis spermatic anomalies. Diseases of the female reproductive system include diseases of the perineum, vagina, uterus, endometrium, cervix, and fallopian tubes [161,162,163]. At present, hydrogels as sustained-release drug carriers are rarely used in male reproductive system diseases, but are more widely used in female reproductive system diseases. Fungal infection is one of the main causes of vaginitis in women. If not treated in time, it can cause further diseases such as cervical disease and endometritis. A previous study showed that nanometer silver antibacterial hydrogel can be used in the treatment of female vaginitis. Because the curative effect is distinct, it also has a good slow-release effect [164,165]. Nanosilver antibacterial hydrogels could also be used for the clinical treatment of female cervical erosion, and the therapeutic effect was significantly better than that of the control group without postoperative treatment [166]. Uterine adhesion is a common disease that occurs after cavity surgery in women, and it often recurs after treatment. Clinically, uterine adhesion can be largely prevented; for example, a hyaluronic acid hydrogel can be used to effectively prevent uterine adhesions after abortion surgery [167]. In addition, chitosan–heparin hydrogel loaded with recombinant human stromal cell-derived factor-1α (SDF-1α) could be used for the repair of injured endometrium to prevent uterine adhesion [168]. The study showed that the hydrogel could promote the repair of endometrium via sustained release of SDF-1α, achieving the effect of preventing uterine adhesion.

## 6. Conclusions and Prospects

At present, the research and development of pharmaceutical preparations is developing toward “three characteristics” (quick effect, high efficiency, and long effect), “three effects” (low toxicity, low dose, and few side-effects), and “five conveniences” (easy production, storage, transportation, carrying, and taking) as important carriers of controlled-release drug systems. They have many advantages, such as a high biocompatibility, low toxicity, simple preparation method, and intelligent regulation, and they are playing increasingly important roles in the field of sustained-release drugs [121,169,170]. In the field of tissue engineering, hydrogels can be used as sustained-release carriers of drugs or applied in tissue diseases by making them into scaffolds. However, most of these are still at the stage of animal experimentation, and there is still a long way to go before they can be widely applied.

Today, the development of hydrogels generally presents a trend towards making them intelligent, harmless, and complex; however, in this process, some serious problems have been exposed; e.g., how to accurately control the physical and chemical properties of a hydrogel in terms of technology to achieve space-time regulation [171], how to realize dynamic intelligent drug delivery in the animal body according to the dynamic changes of the animal environment, how to produce better hydrogels without using potentially toxic crosslinking agents, and how to summarize the advantages of various materials as easily as possible. In terms of practical application, some responsive hydrogels, such as cellulose-based environment-responsive hydrogels, have a low short-term application maturity, relatively concentrated research field, and limited extensiveness. Moreover, these materials are industrially produced on a small scale. In terms of the cost of production, chondroitin sulfate, for example, has good histocompatibility with the body and wide application in tissue engineering; however, due to its relatively singular source, its production cost is high, greatly limiting the use of this high-quality raw material as a drug carrier.

In future studies, the research and development of advanced processing technology, the identification of new materials, and multidisciplinary collaboration, including mechanics, will be important directions in which to solve these types of problems. We hope that, through this approach, the application of hydrogels as sustained-release carriers of drugs in tissue engineering can be further expanded to better benefit mankind.

## Data Availability

Data are contained within the article.
